# Supplementary material for: Genetic profiling of inherited colorectal cancer syndromes in Tunisian patients
Source: PLoS One. 2025 Jun 24;20(6):e0326343. doi: 10.1371/journal.pone.0326343 (PMC12186982; doi:10.1371/journal.pone.0326343)
Supplement: Attachment — Submitted filename: response to reviewers.docx [file pone.0326343.s003.docx]

When submitting your revision, we need you to address these additional requirements.

1. Please ensure that your manuscript meets PLOS ONE's style requirements, including those for file naming. The PLOS ONE style templates can be found at https://journals.plos.org/plosone/s/file?id=wjVg/PLOSOne_formatting_sample_main_body.pdf and https://journals.plos.org/plosone/s/file?id=ba62/PLOSOne_formatting_sample_title_authors_affiliations.pdf

2. PLOS requires an ORCID iD for the corresponding author in Editorial Manager on papers submitted after December 6th, 2016. Please ensure that you have an ORCID iD and that it is validated in Editorial Manager. To do this, go to ‘Update my Information’ (in the upper left-hand corner of the main menu), and click on the Fetch/Validate link next to the ORCID field. This will take you to the ORCID site and allow you to create a new iD or authenticate a pre-existing iD in Editorial Manager.

The ORCID of the corresponding author was added: https://orcid.org/0000-0003-1319-0061

3. Thank you for stating the following in your Competing Interests section:

No

Please complete your Competing Interests on the online submission form to state any Competing Interests. If you have no competing interests, please state "The authors have declared that no competing interests exist.", as detailed online in our guide for authors at http://journals.plos.org/plosone/s/submit-now

This information should be included in your cover letter; we will change the online submission form on your behalf.

4. Thank you for stating the following financial disclosure:

Tunisian Ministry of Higher education and Scientific Research

Please state what role the funders took in the study. If the funders had no role, please state: "The funders had no role in study design, data collection and analysis, decision to publish, or preparation of the manuscript."

If this statement is not correct you must amend it as needed.

Please include this amended Role of Funder statement in your cover letter; we will change the online submission form on your behalf.

The statement was modified in the manuscript and added to the cover letter as recommended

5. Your ethics statement should only appear in the Methods section of your manuscript. If your ethics statement is written in any section besides the Methods, please move it to the Methods section and delete it from any other section. Please ensure that your ethics statement is included in your manuscript, as the ethics statement entered into the online submission form will not be published alongside your manuscript

6. We note that you have indicated that there are restrictions to data sharing for this study. For studies involving human research participant data or other sensitive data, we encourage authors to share de-identified or anonymized data. However, when data cannot be publicly shared for ethical reasons, we allow authors to make their data sets available upon request. For information on unacceptable data access restrictions, please see http://journals.plos.org/plosone/s/data-availability#loc-unacceptable-data-access-restrictions.

Before we proceed with your manuscript, please address the following prompts:

a) If there are ethical or legal restrictions on sharing a de-identified data set, please explain them in detail (e.g., data contain potentially identifying or sensitive patient information, data are owned by a third-party organization, etc.) and who has imposed them (e.g., a Research Ethics Committee or Institutional Review Board, etc.). Please also provide contact information for a data access committee, ethics committee, or other institutional body to which data requests may be sent.

b) If there are no restrictions, please upload the minimal anonymized data set necessary to replicate your study findings to a stable, public repository and provide us with the relevant URLs, DOIs, or accession numbers. Please see http://www.bmj.com/content/340/bmj.c181.long for guidelines on how to de-identify and prepare clinical data for publication. For a list of recommended repositories, please see https://journals.plos.org/plosone/s/recommended-repositories. You also have the option of uploading the data as Supporting Information files, but we would recommend depositing data directly to a data repository if possible.

Please update your Data Availability statement in the submission form accordingly.

7. In the online submission form, you indicated that data are available from the corresponding authoy upon request.

All PLOS journals now require all data underlying the findings described in their manuscript to be freely available to other researchers, either 1. In a public repository, 2. Within the manuscript itself, or 3. Uploaded as supplementary information.

This policy applies to all data except where public deposition would breach compliance with the protocol approved by your research ethics board. If your data cannot be made publicly available for ethical or legal reasons (e.g., public availability would compromise patient privacy), please explain your reasons on resubmission and your exemption request will be escalated for approval.

8. Please amend your authorship list in your manuscript file to include author Dorra Ben Ayed Guerfali

Dorra Ben Ayed Guerfali was included

9. Please amend the manuscript submission data (via Edit Submission) to include author Salah Boujelbene.

Yes it was edited

10. Please include your tables as part of your main manuscript and remove the individual files. Please note that supplementary tables (should remain/ be uploaded) as separate "supporting information" files.

Tables were inserted in the main manuscript as recommended

Reviewers' comments:

Reviewer's Responses to Questions

Comments to the Author

1. Is the manuscript technically sound, and do the data support the conclusions?

The manuscript must describe a technically sound piece of scientific research with data that supports the conclusions. Experiments must have been conducted rigorously, with appropriate controls, replication, and sample sizes. The conclusions must be drawn appropriately based on the data presented.

Reviewer #1: Yes

Reviewer #2: Partly

2. Has the statistical analysis been performed appropriately and rigorously?

Reviewer #1: Yes

Reviewer #2: N/A

3. Have the authors made all data underlying the findings in their manuscript fully available?

The PLOS Data policy requires authors to make all data underlying the findings described in their manuscript fully available without restriction, with rare exception (please refer to the Data Availability Statement in the manuscript PDF file). The data should be provided as part of the manuscript or its supporting information, or deposited to a public repository. For example, in addition to summary statistics, the data points behind means, medians and variance measures should be available. If there are restrictions on publicly sharing data—e.g. participant privacy or use of data from a third party—those must be specified.

Reviewer #1: No

Reviewer #2: Yes

4. Is the manuscript presented in an intelligible fashion and written in standard English?

PLOS ONE does not copyedit accepted manuscripts, so the language in submitted articles must be clear, correct, and unambiguous. Any typographical or grammatical errors should be corrected at revision, so please note any specific errors here.

Reviewer #1: Yes

Reviewer #2: Yes

5. Review Comments to the Author

Please use the space provided to explain your answers to the questions above. You may also include additional comments for the author, including concerns about dual publication, research ethics, or publication ethics. (Please upload your review as an attachment if it exceeds 20,000 characters)

**Point by point response to the reviewers**

Reviewer #1: In this manuscript the authors confirmed 17 of 30 Tunisian colorectal cancer patients by identifying pathogenic variants using Sanger sequencing or Targeted NGS with a 31-gene panel. Results highlighted the necessity of genetic testing in confirming the genetic causes of CRC syndromes, especially in Lynch Syndrome. Minor issues:

1, Page 7 in the Results section, “five teen patients” should be fifteen patients.

It was corrected in the Result section

2, Inconsistency of labeling: in figure 2a, “MP3” should be “MP13”. In addition, In page 7, patients were referred as MAP13, MAP14, MAP15 but in page 10 and in figure 2 they were labelled as MP13, MP14, MP15.

In page 7, MAP13, MAP14, MAP15 were replaced by MP13, MP14, MP15 as recommended.

In the figure 2a, it is really the patient MP3 (pedigree and sanger sequencing chromatogram) showed as an example for MAP patients for whom we identified homozygous mutation in the MUTYH gene.

Figure 2b shows patient MP13 (pedigree and sanger sequencing chromatogram) who is heterozygous composite of the 2 variants: c.1143_1144dupGG/c.1103G>A

Figure 2c shows patient MP15 (pedigree and sanger sequencing chromatogram) for whom we identified heterozygous c.1143_1144dupGG in the MUTYH gene

Figure 2 was revised

3, In page 5, the extra dot should be removed in “. Multiplex Ligation-Dependent Probe Amplification”.

It was corrected

4, The NGS sequencing data such as raw reads and SAM files should be deposited to a public repository.

As recommended the fast Q files were deposited in NCBI with the accession number “PRJNA1258780”. We noticed that NGS was performed on 15 samples, and 15 were analysed by sanger sequencing (13 MAP and 2 FAP patients).

5, Since this study focused on CRC samples, it would be better if authors discussed why the OncoRisk Cancer Panel was used instead of targeted CRC panel which typically includes other classical CRC oncogenes such as BRAF, KRAS and CTNNB1.

We totally agree with this comment. Our study focusing on hereditary CRC, we though that the use of the OncoRisk cancer panel would provide a larger and more comprehensive approach than a CRC-specific panel. It is particularly valuable for uncovering non-classical or overlapping hereditary cancer syndromes, guiding family counselling, and expanding our understanding of cancer susceptibility genetics in Tunisian patients

In fact, OncoRisk Cancer Panel is a Pan-cancer panel designed to assess germline variants across a wide range of cancer susceptibility genes, including those not traditionally associated with CRC. This often includes high- and moderate-penetrance genes like *MLH1, MSH2, MSH6, PMS2, APC*, but also genes linked to other cancers such as *BRCA1/2, TP53, CDH1, PTEN*…

Since we are investigating samples with Hereditary CRC that are mainly caused by germline mutations in MMR genes or APC gene, a hereditary-focused panel such as the oncorisk allow us to detect, not only variants in classical CRC gene, but also screening beyond CRC-specific genes to identify pleiotropic cancer predisposition syndromes. In fact, the OncoRisk panel allows detection of unexpected germline mutations that may modify cancer risk management (e.g., finding a BRCA mutation in a CRC patient, which might be relevant for family members or personal risk of other cancers).

Reviewer #2: This study assessed the impact of genetic testing in the diagnosis of 30 patients suspected with hereditary polyposis or non-polyposis syndromes.

1. The authors should clarify the criteria/guidelines used to consider patients as suspected of having hereditary polyposis or non-polyposis syndromes. For example, did the authors follow NCCN Guidelines for Genetic/Familial High-Risk Assessment?

The NCCN (National Comprehensive Cancer Network) guidelines were used for our patient’s selection since they are essential for identifying individuals who may benefit from genetic testing and counseling due to their familial or hereditary cancer risk.

For Lynch Syndrome patients, we used the following criteria:

Personal History: CRC diagnosed before age 50; Endometrial cancer diagnosed before age 50 ; Multiple Lynch syndrome-associated cancers (CRC, endometrial, ovarian, stomach, small bowel, hepatobiliary, upper urinary tract) ; Second CRC or other Lynch-associated cancer within 5-10 years of the first cancer diagnosis.

Family History: At least 3 family members with Lynch-associated cancers (CRC, endometrial cancer, small bowel cancer) ; Cancer diagnoses should be spread across at least 2 generations, and one should be diagnosed before age 50.

For patients with Polyposis syndromes, we used those criteria:

Early age of onset of polyposis; Diagnosis of numerous adenomatous polyps in the colon and rectum; Familial history of polyposis

For other familial Colorectal Cancer (without Defined Syndrome), we focused on the Family History:

Two or more first-degree relatives with CRC or advanced adenomas (increased risk for CRC) ; Diagnosis of CRC in family members at <50 years of age.

For more clarity, we added in the revised manuscript in Material and Methods section p.5 the following sentences:

“according to the NCCN (National Comprehensive Cancer Network) guidelines. Briefly, patients with Polyposis syndromes, the criteria were: early age of onset of polyposis; diagnosis of numerous adenomatous polyps in the colon and rectum, and familial history of polyposis. For Lynch Syndrome patients, the criteria were: CRC and/or endometrial cancer diagnosed before age 50; other Lynch Syndrome-associated cancers such as ovarian, stomach, small bowel, hepatobiliary, upper urinary tract cancers, family history with at least 3 members with Lynch-associated cancers; diagnosis of cancer should be spread across at least 2 generations, and one should be diagnosed before age 50. For other familial Colorectal Cancer (without defined syndrome), we focused on family history with at least two first-degree relatives with CRC or advanced adenomas at <50 years of age.”

2. It would be helpful to include additional data on the concordance between Sanger sequencing and targeted NGS. Specifically, as both Sanger sequencing and targeted NGS were performed for patients MAP14 and MAP15, did the targeted NGS detect the same MUTYH variant identified by Sanger sequencing?

All the variant identified by NGS were confirmed by sanger sequencing and data was included in the figures. For patients MAP14 and MAP15, the Sanger sequencing detected the same MUTYH variant that was identified by targeted NGS.

3. I couldn’t find Table 1 and Table 2.

Tables were included in the manuscript file.

4. The authors should carefully proofread the manuscript to correct typos. Below are a few noted:

Abstract, “hereditary polyposis syndromes such as MUTY-Associated Polyposis” should be “… MUTYH…”

Page 6, “. Multiplex Ligation-Dependent Probe Amplification” should be “Multiplex Ligation-Dependent Probe Amplification”

Page 7, “In this group, five teen patients suspected of MYH-Associated Polyposis” should be “… fifteen…”

We have carefully proofread the manuscript and corrected all identified errors.

6. PLOS authors have the option to publish the peer review history of their article (what does this mean?). If published, this will include your full peer review and any attached files.

If you choose “no”, your identity will remain anonymous but your review may still be made public.

Do you want your identity to be public for this peer review? For information about this choice, including consent withdrawal, please see our Privacy Policy.

Reviewer #1: No

Reviewer #2: No

[NOTE: If reviewer comments were submitted as an attachment file, they will be attached to this email and accessible via the submission site. Please log into your account, locate the manuscript record, and check for the action link "View Attachments". If this link does not appear, there are no attachment files.]

While revising your submission, please upload your figure files to the Preflight Analysis and Conversion Engine (PACE) digital diagnostic tool, https://pacev2.apexcovantage.com/. PACE helps ensure that figures meet PLOS requirements. To use PACE, you must first register as a user. Registration is free. Then, login and navigate to the UPLOAD tab, where you will find detailed instructions on how to use the tool. If you encounter any issues or have any questions when using PACE, please email PLOS at figures@plos.org. Please note that Supporting Information files do not need this step.

In compliance with data protection regulations, you may request that we remove your personal registration details at any time. (Remove my information/details). Please contact the publication office if you have any questions.

Finally, we thank the reviewers for their valuable comments and we hope we answered them satisfactorily.

Best Regards

The corresponding author
